# *KCS1* and *VIP1* , the genes encoding yeast phosphoinositol pyrophosphate synthases, are required for Ca^2+^‐mediated response to dimethylsulfoxide (DMSO)

**DOI:** 10.1002/2211-5463.70039

**Published:** 2025-04-11

**Authors:** Larisa Ioana Gogianu, Lavinia Liliana Ruta, Claudia Valentina Popa, Simona Ghenea, Ileana Cornelia Farcasanu

**Affiliations:** ^1^ Doctoral School of Biology, Faculty of Biology University of Bucharest Romania; ^2^ National Institute for Research and Development in Microtechnologies Voluntari Romania; ^3^ Faculty of Chemistry University of Bucharest Romania; ^4^ Institute of Biochemistry of the Romanian Academy Bucharest Romania

**Keywords:** aequorin, calcium, dimethylsulfoxide, inositol pyrophosphate synthase, *Saccharomyces cerevisiae*

## Abstract

Dimethylsulfoxide (DMSO) is widely used as a solvent or as a carrier when screening for biologic activity of various chemicals, but results need to be interpreted carefully due to its intrinsic toxicity. DMSO has been previously observed to impair the growth of yeast cells defective in calcium movement across cellular membranes and in phosphoinositol pyrophosphate synthases. Here, we set out to investigate the Ca^2+^‐mediated response to DMSO in *Saccharomyces cerevisiae*. The cell exposure to DMSO was signaled by a two‐phase cytosolic Ca^2+^ wave that was dependent on Mid1, a subunit of the Cch1/Mid1 Ca^2+^ channel located at the plasma membrane. While the vacuolar Ca^2+^ channel Trpy1 also contributed by releasing Ca^2+^ from the vacuole, the immediate cell response to DMSO exposure depended on the external Ca^2+^ imported into the cell through Cch1/Mid1. A chemogenomic screen previously performed on a collection of yeast knockout mutants identified the two phosphoinositol pyrophosphate synthases Kcs1 and Vip1 as determinants for yeast tolerance to DMSO. Deletion of *KCS1* or *VIP1* genes suppressed the DMSO‐induced Ca^2+^ response, suggesting that both Ca^2+^ and phosphoinositol pyrophosphate signaling contribute to cell adaptation under DMSO stress.

## Graphical abstract

Ca^2+^‐mediated response to DMSO was investigated in *Saccharomyces cerevisiae* cells expressing Ca^2+^‐dependent aequorin. Cell exposure to DMSO induced a cytosolic Ca^2+^ wave dependent on the integrity of the Cch1/Mid1 channel. Deletion of *KCS1* or *VIP1* genes encoding the phosphoinositol pyrophosphate (PP‐IP) synthases suppressed the DMSO‐induced Ca^2+^ response, indicating that both Ca^2+^ and PP‐IP signaling contribute to cell adaptation under DMSO stress.

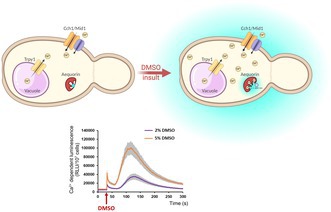

**Abbreviations**
1,5PP‐IP4: 1,5‐bis‐diphosphoinositol tetrakisphosphate
1PP‐IP5: 1‐diphosphoinositol pentakisphosphate
5PP‐IP4: 5‐diphosphoinositol tetrakis phosphate
5PP‐IP5: 5‐diphosphoinositol pentakisphosphate
BAPTA: *O,O′*‐bis (2‐aminophenyl) ethyleneglycol‐*N,N,N′,N′*‐tetraacetic acid
[Ca^2+^]_cyt_
: cytosolic calcium
DMF: dimethylformamide
DMSO: dimethylsulfoxide
IC_50_
: half inhibitory concentration
IP6: inositol‐hexakisphosphate
IP6K: inositol‐hexakisphosphate kinase
PP‐IP: phosphoinositol pyrophosphate (diphosphate)
SD: synthetic dextrose medium
SD‐Ura: synthetic dextrose medium lacking uracil
SEM: standard error of the mean
TPI: triose‐phosphate isomerase
WT: wild‐type (parental type)
YPD: yeast extract‐peptone‐dextrose medium

Dimethylsulfoxide (DMSO) is a polar nonprotic solvent widely used in biochemistry, biotechnology, and pharmacological studies as an important adjuvant to compounds that are not soluble in water [1]. DMSO has certain traits that strongly recommend it as a solvent for the compounds tested in biological screenings and cellular assays: It has strong polarity, it is amphiphilic, being miscible with both water and organic phases, and it has low chemical reactivity [2]. DMSO is also used as a radio‐ and cryo‐protectant, or as a drug delivery agent due to its potential to interact with the plasma membrane [3] through mechanisms that are not fully elucidated [4]. Although widely used as a solvent or as a carrier when screening for biologic activity of various chemicals, the outcomes need to be considered with care, due to the potential overlapping or misinterpretation caused by the eventual DMSO's intrinsic toxicity [5, 6, 7].

The budding yeast *Saccharomyces cerevisiae* is one of the eukaryotic microorganisms often employed for the development of genomic approaches used for the screening of chemicals' interaction with the living cells. Specifically, with the availability of genome‐wide collections of individual knockout mutants, it serves as an effective tool for screening of compounds' toxicity [8, 9, 10]. In a previous study, we used a set of approximately 4500 haploid *S. cerevisiae* single‐gene knockout mutants to identify the potential targets of a copper(II) methionine‐derived complex compound [11]. Since the tested compound was used from a DMSO stock solution, a control was set in which the cells were grown in the presence of DMSO only. It was noted that in the cases of some knockout mutants, the apparent toxicity of the compound overlapped the toxicity of the solvent itself. Notable strains that exhibited decreased tolerance to DMSO belonged to two groups: (a) Mutants defective in calcium transport across membranes, or (b) mutants lacking either of the two yeast phosphoinositol pyrophosphate (PP‐IP) synthases, Kcs1 and Vip1. As both calcium and phosphoinositol pyrophosphates are important messengers within the cell, we decided to check if DMSO exposure triggers a Ca^2+^‐mediated response and if the activity of PP‐IP synthases interferes with Ca^2+^‐mediated response to DMSO.

Like most living organisms, *S. cerevisiae* cells respond to a variety of changes in the environment through signaling pathways that utilize Ca^2+^ as a second messenger. Ca^2+^‐mediated responses have been reported for yeast cells exposed to high and low osmolarity [12, 13], salt stress [12], high alkalinity [14], cytotoxic compounds [15, 16], oxidative stress [17], ethanol stress [18], or heavy metal stress [19, 20], and they are mainly achieved by triggering an abrupt increase in cytosolic calcium ([Ca^2+^]_cyt_) that enters the cell through the Mid1/Cch1 plasma membrane channel [12, 21, 22]. Additional to its external provenance, Ca^2+^ can be released into the cytosol from the vacuole via the Trpy1 channel (initially named Yvc1), release that is often stimulated by the entry of external Ca^2+^ into the cytosol [17, 22]. To ensure the abrupt increase of [Ca^2+^]_cyt_, reciprocal regulation is possible, depending on Ca^2+^ availability. Specifically, the release of vacuolar Ca^2+^ via Trpy1 can be stimulated by the Ca^2+^ from outside the cell, as well as by Ca^2+^ released from the vacuole by Trpy1 itself, in a positive feedback called Ca^2+^‐induced Ca^2+^ release (CICR) [23, 24, 25, 26]. Vice versa, the release of Ca^2+^ from intracellular stores stimulates the extracellular Ca^2+^ influx, a process known as capacitative Ca^2+^ entry [27]. In both cases, once the message is delivered, the [Ca^2+^]_cyt_ has to be restored to the normally very low concentration through the action of Ca^2+^ pumps and exchangers, that is, the vacuolar Ca^2+^‐ATPase Pmc1 and the vacuolar Ca^2+^/H^+^ exchanger Vcx1 (which independently transport [Ca^2+^]_cyt_ into the vacuole) and/or the secretory Ca^2+^‐ATPase Pmr1, which pumps [Ca^2+^]_cyt_ into the endoplasmic reticulum (ER) and Golgi [28].

Proteins Kcs1 and Vip1 are the enzymes responsible for the synthesis of phosphoinositol pyrophosphates (PP‐IPs) in yeast, and the only yeast enzymes known to have inositol hexakis‐phosphate kinase (IP6K) activity (for a recent review, [29]). PP‐IPs are signaling molecules that orchestrate many cellular processes in yeast and higher organisms [30, 31, 32, 33, 34, 35, 36, 37], including response to various stresses [38]. Kcs1 forms a pyrophosphate (diphosphate) by adding a phosphate to the 5th position of the inositol polyphosphate ring, thus participating in the synthesis of various inositol pyrophosphates, such as 5‐diphosphoinositol pentakisphosphate (5PP‐IP5), 1,5‐bis‐diphosphoinositol tetrakisphosphate (1,5PP‐IP4), and 5‐diphosphoinositol tetrakisphosphate (5PP‐IP4) [39, 40], a reaction reversed by Siw14, an inositol pyrophosphatase that hydrolyzes the β‐phosphate from 5‐diphospho‐position [41]. Vip1, on the other hand, forms a pyrophosphate (diphosphate) by adding a phosphate to the 1st position of the inositol polyphosphate ring in the synthesis of 1‐diphosphoinositol pentakisphosphate (1PP‐IP5) and 1,5‐bis‐diphosphoinositol tetrakisphosphate (1,5PP‐IP4) [29, 42, 43], an action reversed by either Dipp1 [41] or Vip1 itself [43].

Starting from the observation that DMSO impaired the growth of yeast cells defective in (a) calcium movement across cellular membranes and (b) in phosphoinositol pyrophosphate synthases, we set up a study to reveal the potential correlation between Ca^2+^‐mediated response to DMSO exposure and the two PP‐IP synthases, Kcs1 and Vip1.

## Materials and methods

### Reagents

All chemicals and culture media components were purchased from Merck (Darmstadt, Germany). Dimethylsulfoxide (DMSO) and *N*,*N*‐dimethylformamide (DMF) were of molecular biology grade (purity > 99%) and were filter‐sterilized before use (Millipore syringe filters, 0.22 μm pores, Merck, Darmstadt, Germany).

### Yeast strains and media

The *S. cerevisiae* parental strain (considered wild‐type, WT) was BY4741 (*MAT*a *his3Δ1*; *leu2Δ0*; *ura3Δ0*), a S288C‐based yeast [44]. The isogenic knockout strains with individual gene deletions were *cch1Δ* (BY4741 *cch1::kanMX4*), *mid1Δ* (BY4741 *mid1::kanMX4*), *trpy1Δ* (BY4741 *trpy1::kanMX4*), *kcs1Δ* (BY4741 *kcs1::kanMX4*), and *vip1Δ* (BY4741 *vip1::kanMX4*). The strains were obtained from EUROSCARF (http://www.euroscarf.de/). Yeast cells were maintained, grown and propagated in YPD medium (1% w/v yeast extract, 2% w/v polypeptone, 2% w/v glucose) or in synthetic dextrose complete (SD) medium (0.67% w/v yeast nitrogen base, 2% w/v glucose supplemented with the necessary amino acids) [45]. For solid media, 2% agar was used. When used in growth‐related experiments, filter‐sterilized DMSO was added to autoclaved media cooled to 55 °C.

### Yeast plasmids and transformation

Plasmids pYX212 (*URA3*‐based, triose phosphate isomerase TPI promoter) and pYX212‐cytAEQ harboring aequorin cDNA [46] were a generous gift from E. Martegani and R. Tisi (University of Milano‐Bicocca, Milan, Italy). Plasmids pYX212‐KCS1 and pYX212‐VIP1 were obtained by introducing the PCR‐amplified coding sequences of *KCS1* and *VIP1* in the multicloning site of pYX212, downstream of the TPI promoter. The *KCS1* and *VIP1* open reading frames were amplified by PCR (GoTaq® G2 PCR Master Mix; Promega, Madison WI, USA) from genomic DNA isolated from strain BY4741 with the Wizard® Genomic DNA Purification Kit (Promega). Primers used for amplification were: for *KCS1*, forward: 5′‐gaaccATGGATACCTCTCACGAAATTCATG, reverse: 5′‐gaactcgagTCAATCACTAACTTGAGCATCGTC; for *VIP1* forward: 5′‐gaaccatggGT GGG ATAAAGAAGGAACCGATTG and reverse: 5′‐gaactcgagCTAATCTAATGTCTTGTTAACGGAGG. The lowercase letters denote sequences not extant naturally, introduced for cloning purposes. The forward and reverse primers introduced the *Nco*I and *Xho*I sites, respectively (underlined), used to clone the amplicons into the *Nco*I/*Xho*I site of the pYX212 vector. Yeast transformation [47] was performed using the S.c. EasyComp™ Transformation Kit (Invitrogen, Thermo Fisher Scientific, Waltham, MA, USA) following the manufacturer's indications.

### The effect of DMSO on cell growth

Cells from overnight cultures were inoculated in fresh media at a density of 2 × 10^5^ cells·mL^−1^ and grown for 2 h before adding sterile DMSO at various concentrations (considered time 0). All throughout the experiments, cells were incubated with shaking (200 r.p.m.) at 30 °C. Strain growth was monitored by measuring the turbidity of cell cultures at 600 nm (OD_600_) recorded by a plate reader equipped with a thermostat and a shaker (Varioskan, Thermo Fisher Scientific, Vantaa, Finland). Relative growth was expressed as the ratio between OD_600_ recorded at time *t* and OD_600_ recorded at time 0 for each individual strain.

### Detection of [Ca^2+^]_cyt_ fluctuations

Cells expressing functional aequorin were prepared for [Ca^2+^]_cyt_ detection as described [46], with slight modifications. Cells transformed with pYX212‐AEQ harboring apo‐aequorin cDNA were inoculated from an overnight culture in SD‐Ura and grown (200 r.p.m., 30 °C) to OD_600_ = 1, then concentrated by centrifugation to OD_600_ = 10. To reconstitute functional aequorin, native coelenterazine was added to the cell suspension (from a methanol stock, 20 μm final concentration) and incubated for 2 h at 30 °C in the dark. Cells were subsequently washed to remove the excess coelenterazine and re‐suspended in SD‐Ura supplemented with 2 mm CaCl_2_. The cells were transferred (approximately 10^7^ cells/determination) to the luminometer tube and a cellular luminescence baseline was determined for each strain by approximately 1 min of recordings at 1/s intervals. After ensuring a stable signal, DMSO was injected under strong agitation. To induce oxidative or alkaline stress, H_2_O_2_ and KOH were injected to final concentrations of 5 and 10 mm, respectively. The added KOH changed the pH of the cell environment from 5.8 to approximately 8.5. The Ca^2+^‐dependent light emission was monitored in a single tube luminometer (20n/20; Turner Biosystems, Sunnyvale, CA, USA). The light emission was measured at 1‐s intervals and expressed as relative luminescence units (RLU). To mimic Ca^2+^ depletion, the Ca^2+^ chelator *O,O′*‐bis (2‐aminophenyl) ethyleneglycol‐*N,N,N′,N′*‐tetraacetic acid (BAPTA) was added 30–45 s prior to DMSO exposure at 5 mm final concentration. BAPTA concentration surpassed the Ca^2+^ concentration in the media, ensuring complete chelation. To ensure that the total reconstituted aequorin was not limiting in our assay, at the end of each experiment aequorin remnant activity was checked by lysing cells with 14% Triton X‐100 with 5 mm CaCl_2_; only the cells with considerable residual luminescence were considered. Relative luminescence emission was normalized to an aequorin content giving a total light emission of 10^6^ RLUs in 10 min after lysing cells with 14% Triton X‐100.

### Statistics

Experiments were repeated independently in at least three biological replicates and only the experimental setups that yielded similar trends were considered. For each individual experiment, values were expressed as the mean ± standard error of the mean (SEM). For aequorin luminescence determinations, traces represent the mean ± SEM from three independent transformants. The numerical data were examined by one‐sample *t‐*test or by analysis of variance with multiple comparisons (ANOVA) using the statistical software prism version 6.05 for Windows (GraphPad Software, La Jolla, CA, USA). The differences were considered significant for *P* < 0.05.

## Results

### DMSO affects yeast cell proliferation

**Fig. 1:**
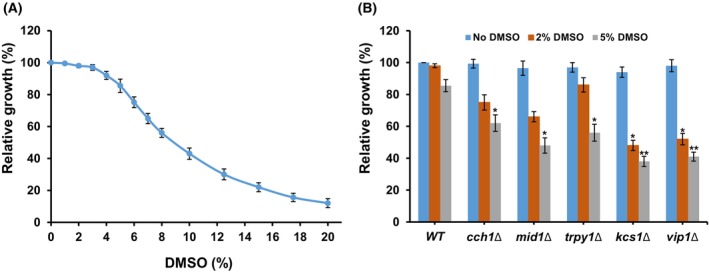
Effect of dimethyl sulfoxide (DMSO) on yeast growth. (A) Effect of DMSO concentration on growth of wild‐type cells. (B) Growth of DMSO‐sensitive mutants. Yeast cells (wild‐type and knockout mutants) were inoculated from exponentially growing YPD cultures in SD liquid medium (2 × 10^5^ cells·mL^−1^ initially) in the presence of various concentrations of DMSO and incubated with shaking (200 r.p.m.) at 30 °C. Cell density was determined spectrophotometrically at 600 nm (OD_600_). Relative growth was determined as described in the Materials and methods section 24 h after the addition of DMSO and expressed as mean ± SEM (*n* = 3). Two‐way ANOVA with Bonferroni post‐test showing the level of significance when comparing each mutant with wild‐type strain exposed to the same concentration of DMSO. *P < 0.05; **P < 0.01.

A screen previously performed on a collection of *S. cerevisiae* knockout mutants to identify the potential targets of a methionine‐Cu(II) coordinative compound [11] indicated that in some cases, the apparent toxicity of the compound was caused by the intrinsic action of the solvent in which the working stock solution was prepared, that is, DMSO. The growth of the parental yeast cells (considered wild‐type, WT) exposed to various concentrations of DMSO was therefore determined, and it was found that cell proliferation was affected by DMSO in a concentration‐dependent manner (Fig. 1A). When present in the incubation medium in 1–3% concentration, the growth of the WT cells was not significantly affected (cells exhibited > 95% of the growth in the normal conditions). At 5% DMSO concentration, the growth of WT cells was approximately 85% of the growth under normal conditions, while 8–10% DMSO inhibited the growth by approximately 50% (calculated half inhibitory concentration, IC_50_ = 8.52). More sensitive to DMSO exposure were the mutants that were defective in plasma membrane (*cch1Δ* and *mid1Δ*) or vacuole membrane (*trpy1Δ*) Ca^2+^ channels, but also in phosphoinositol‐pyrophosphate synthases (*kcs1Δ* and *vip1Δ*) (Fig. 1B).

### DMSO triggers Ca^2+^ entry into the cell

**Fig. 2:**
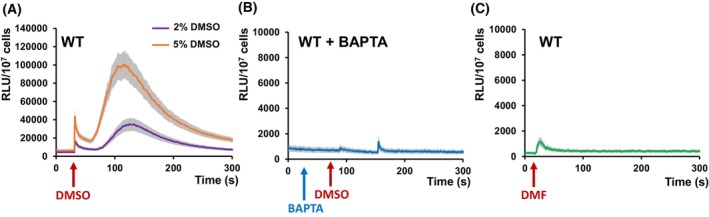
Dimethyl sulfoxide (DMSO) exposure induces the increase of cytosolic Ca^2+^ ([Ca^2+^]_cyt_). Wild‐type (WT) cells expressing reconstituted aequorin were pregrown in SD‐Ura and subjected to DMSO stress as described in the Materials and methods section. [Ca^2+^]_cyt_‐dependent aequorin luminescence was recorded on samples of approximately 10^7^ cells (OD_600_ = 1). The arrows indicate the moment when the chemicals were added to induce stress. (A) Ca^2+^‐dependent luminescence of wild‐type exposed to DMSO. (B) Inhibition of Ca^2+^‐dependent luminescence by Ca^2+^ chelator BAPTA (5 mm final concentration). BAPTA was added 45 s prior to DMSO exposure (5% final concentration). (C) Dimethylformamide (DMF) does not induce Ca^2+^‐mediated luminescence. The curve shows the response to 5% DMF (final concentration) and no Ca^2+^‐dependen response could be recorded for other lower or higher concentrations. The luminescence traces represent the mean ± SEM from three independent transformants. RLU, relative luminescence units.

The mutants *cch1Δ* and *mid1Δ* were more sensitive to DMSO than the WT cells (Fig. 1B). Since *CCH1* and *MID1* genes encode for the two subunits of the Ca^2+^ ion channel Cch1/Mid1 located at the plasma membrane, we wondered if DMSO exposure triggers a Ca^2+^ pulse into the cytosol, as in the case of other environmental stresses [12, 13, 14, 15, 16, 17, 18, 19, 20]. To test this possibility, we monitored the fluctuations of [Ca^2+^]_cyt_ in cells expressing the Ca^2+^ photoprotein reporter, aequorin [48]. For this purpose, the tested cells were transformed with apo‐aequorin cDNA situated under the control of a constitutive TPI promoter that allows high expression of the photoprotein in the cytosol [49]. Transgenic cells overexpressing apo‐aequorin were incubated with the cofactor coelenterazine to yield the functional aequorin; further, any increase in [Ca^2+^]_cyt_ would be detected as an increase in cell luminescence. It was noted that the exposure of WT cells expressing functional aequorin to DMSO resulted in two‐phase Ca^2+^‐dependent luminescence, that is: (a) one sharp and fast response, with a peak recorded within seconds after DMSO addition; (b) a more gradual response, with a maximum intensity recorded after 120–125 s after DMSO addition, and with a return to the almost basal luminescence after 250–300 s (Fig. 2A). The intensity of the response depended on the concentration of DMSO applied to the cell (Fig. 2A). The lowest concentration that generated a recordable response was around 2%. The two‐phase luminescence trace was no longer visible when the DMSO‐exposed cells were pretreated with the Ca^2+^ chelator BAPTA (Fig. 2B), indicating that the presence of DMSO in the environment is sensed by the yeast cells through a Ca^2+^ wave that originates from the exterior of the cell. To avoid intracellular Ca^2+^ depletion, BAPTA was added to the cells' medium 30–45 s prior to DMSO exposure.

The cell response to another solvent widely used in drug testing, dimethylformamide (DMF), was also tested. Nevertheless, DMF elicited no Ca^2+^‐dependent luminescence in WT cells expressing functional aequorin, despite certain structural similarities to DMSO (Fig. 2C).

### The Mid1 component of the Cch1/Mid1 channel is crucial for DMSO‐induced Ca^2+^ entry into the cytosol

**Fig. 3:**
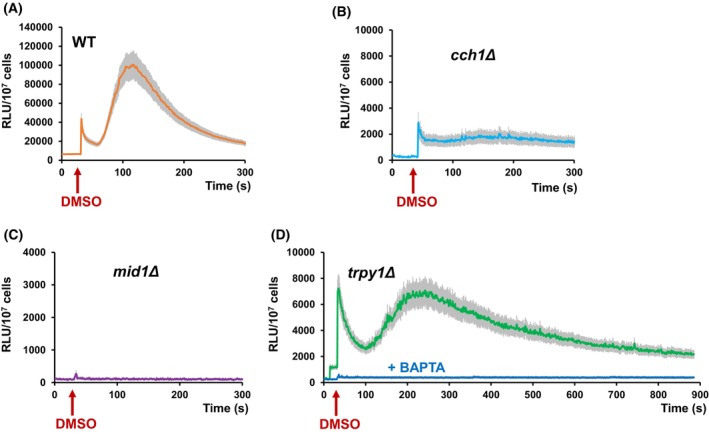
Ca^2+^‐dependent response to dimethyl sulfoxide (DMSO) of cells with defects in Ca^2+^ channels. Wild‐type (WT) and isogenic knockout mutants *cch1Δ*, *mid1Δ*, or *trpy1Δ* expressing reconstituted aequorin were pregrown in SD‐Ura and subjected to DMSO stress as described in the Materials and methods section. [Ca^2+^]_cyt_‐dependent aequorin luminescence was recorded on samples of approximately 10^7^ cells (OD_600_ = 1). The arrows indicate the moment when DMSO was added to achieve a 5% final concentration. Ca^2+^‐dependent luminescence of: (A) wild‐type, (B) *cch1Δ*, (C) *mid1Δ*, or (D) *trpy1Δ* exposed to 5% DMSO. Blue line in D: response of *trpy1Δ* cells pretreated with 5 mm BAPTA 30 s prior to DMSO exposure. The luminescence traces represent the mean ± SEM from three independent transformants. RLU, relative luminescence units.

In the absence of mobile extracellular Ca^2+^ (e.g., Ca^2+^ chelation by BAPTA), no significant increase in [Ca^2+^]_cyt_ was detectable (Fig. 2B), suggesting that in response to DMSO exposure, Ca^2+^ might enter the cytosol across the plasma membrane through the Cch1/Mid1 channel. To test this possibility, the change in [Ca^2+^]_cyt_ was recorded in the mutants *cch1Δ* and *mid1Δ* expressing functional aequorin. Only a faint response was recorded in *cch1Δ* cells expressing aequorin (Fig. 3A). However, it was noted that the transient increase in [Ca^2+^]_cyt_ which follows the addition of DMSO was largely absent in the *mid1Δ* strain (Fig. 3B), indicating that Mid1 plays a pivotal role in Ca^2+^ entry under DMSO stress. As both *cch1Δ* and *mid1Δ* were more sensitive to DMSO than the wild‐type (Fig. 1B), it can be speculated that Ca^2+^ entry into the cytosol is required for cell adaptation to DMSO stress, and that DMSO activates Mid1p for Ca^2+^ influx across the plasma membrane, while Cch1 is necessary for optimal Mid1 activity.

The *trpy1Δ* cells expressing functional aequorin (lacking the channel that releases vacuolar Ca^2+^ into the cytosol) responded to DMSO with a biphasic [Ca^2+^]_cyt_‐dependent luminescence (Fig. 3C) similarly to wild‐type cells. Nevertheless, unlike the wild‐type cells, the luminescence traces were broader and peaked lower, indicating that functional Trpy1 is needed for achieving the required Ca^2+^ pulse under DMSO exposure. Thus, it is likely that Trpy1 contributes to the [Ca^2+^]_cyt_ pool under DMSO stress and that the release of vacuolar Ca^2+^ via Trpy1 is probably stimulated by the Ca^2+^ from outside the cell when DMSO‐induced [Ca^2+^]_cyt_ reaches a critical threshold. In this line of evidence, it was noted that BAPTA pre‐treatment (45 s prior to DMSO addition) completely attenuated the [Ca^2+^]_cyt_ wave in *trpy1Δ* cells (Fig. 3C, blue line).

### Deletion of *KCS1* or *VIP1* affects DMSO‐induced [Ca^2+^]_cyt_ elevations

**Fig. 4:**
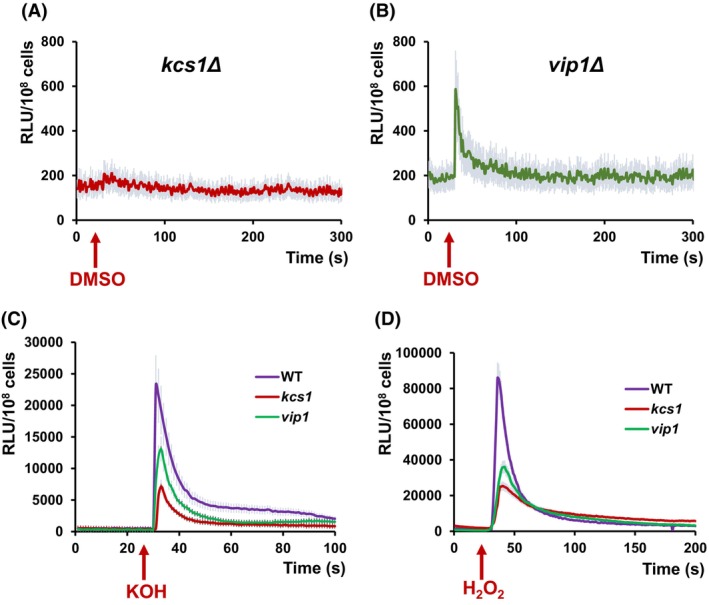
Ca^2+^‐dependent response of cells defective in inositol pyrophosphates (PP‐IPs) synthesis. Wild‐type (WT) and isogenic knockout mutants *kcs1Δ* or *vip1Δ* expressing reconstituted aequorin were pregrown in SD‐Ura and subjected to various stresses as described in the Materials and methods section. [Ca^2+^]_cyt_‐dependent aequorin luminescence was recorded on samples of approximately 10^7^ cells (OD_600_ = 1). The arrows indicate the moment when the stressor was added. (A) Ca^2+^‐dependent luminescence of *kcs1Δ* exposed to 5% DMSO. (B) Ca^2+^‐dependent luminescence of *vip1Δ* exposed to 5% DMSO. (C) Effect of alkaline stress (10 mm KOH, final concentration, pH approximately 8.5) on Ca^2+^‐dependent luminescence of WT, *kcs1Δ*, and *vip1Δ* cells. (D) Effect of oxidative stress (5 mm H_2_O_2_, final concentration) on Ca^2+^‐dependent luminescence of WT, *kcs1Δ*, and *vip1Δ* cells. The luminescence traces represent the mean ± SEM from three independent transformants. RLU, relative luminescence units.

As the cells devoid of either of the two phosphoinositol pyrophosphate synthases Kcs1 or Vip1 were sensitive to DMSO (Fig. 1B), the Ca^2+^ entry into the cytosol under DMSO stress was investigated in the corresponding knockout mutants. It was noted that in *kcs1Δ* cells expressing functional aequorin, the DMSO‐induced Ca^2+^ entry was practically absent (Fig. 4A), while in the case of *vip1Δ* cells only a faint mono‐phasic increase in [Ca^2+^]_cyt_‐dependent luminescence was recorded (Fig. 4B). Both Kcs1 and Vip1 were reported to be involved in ribosome biogenesis [36]; therefore, we wondered whether the lack of response to DMSO exposure could be the result of defective protein biosynthesis and subsequently, of low expression of the transgenic apo‐aequorin in the cytosol. This was not the case, because when permeabilizing the cells with 14% Triton X100, strong luminescence was recorded, indicating that the eventual insufficient apo‐aequorin biosynthesis was not the limiting cause. Moreover, a Ca^2+^‐mediated response to alkaline stress (Fig. 4C) or to oxidative stress imposed by external H_2_O_2_ (Fig. 4D) was also recorded in both *kcs1Δ* and *vip1Δ* cells (although to a lower level compared to the wild‐type), indicative of aequorin functionality.

### Effect of *KCS1* and *VIP1* overexpression on DMSO tolerance

**Fig. 5:**
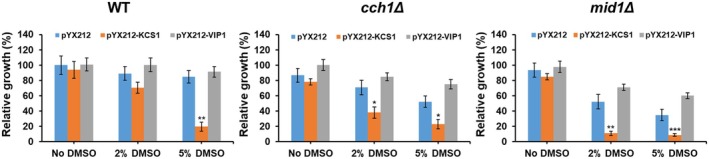
Effect of *KCS1* or *VIP1* overexpression on yeast tolerance to dimethyl sulfoxide (DMSO). WT, *cch1Δ*, and *mid1Δ* cells transformed with pYX212 (empty vector), pYX212‐KCS1 (expressing *KCS1* from TPI promoter) or pYX212‐VIP1 (expressing *VIP1* from TPI promoter) were inoculated from exponentially growing cultures in SD‐Ura liquid medium to an initial density of 2 × 10^5^ cells·mL^−1^ in the presence of various concentrations of DMSO. Cell density was determined spectrophotometrically at 600 nm (OD_600_). Relative growth was determined as described in the Materials and methods section 24 h after the addition of DMSO and expressed as mean ± SEM (*n* = 3). Two‐way ANOVA with Bonferroni posttest indicated the level of significance when comparing each overexpressing mutant with the cells harboring the empty vector at the same concentration of DMSO. *P < 0.05; **P < 0.01; ***P < 0.001.

As *KCS1* and *VIP1* gene deletion resulted in increased sensitivity to DMSO exposure, but also in low Ca^2+^‐mediated luminescence of cells expressing functional aequorin, the effect of *KCS1* and *VIP1* overexpression on the tolerance to DMSO was also determined in both wild‐type and cells devoid of either of the two components of the Cch1/Mid1 channel. For this purpose, both *KCS1* and *VIP1* genes were cloned in the pYX212 vector, which allows gene expression from the constitutive strong TPI promoter. It was noted that *VIP1* overexpression slightly improved or at least did not significantly change the tolerance to DMSO of both WT and *cch1Δ* or *mid1Δ* cells. In contrast, *KCS1* overexpression was deleterious to cells exposed to DMSO, especially to *mid1Δ* cells (Fig. 5).

## Discussions

Understanding the yeast response to DMSO is of utmost importance considering its extensive use in chemical, pharmaceutical, and biomedical applications. We first evaluated the proliferation of wild‐type *S. cerevisiae* exposed to varying concentrations of DMSO. Our results indicated that medium‐to‐high concentrations (2–10%) induce stress, with a calculated IC_50_ of 8.52%. This finding aligns with previous studies, which collectively suggest that exposure to low DMSO inhibits proliferation without necessarily compromising viability, as demonstrated by long‐term survival and recovery assays [50, 51]. Additionally, DMSO exposure triggers a stress response, with its effects depending on concentration and exposure duration. In this study, it was shown that Ca^2+^ plays a role in the cells' response to the stress induced by the presence of DMSO in the cell environment, as seen from the lower tolerance to DMSO of the mutants lacking the genes that code for yeast Ca^2+^ channels. Moreover, the occurrence of the stress‐induced [Ca^2+^]_cyt_ pulse detected in real time using the aequorin assay provided some hints on the role of each channel in modulating the Ca^2+^‐mediated response to DMSO stress. Since the wild‐type cells signaled the presence of DMSO through a strong biphasic [Ca^2+^]_cyt_ wave that primarily originated from external Ca^2+^, it was concluded that the main actor in the response to DMSO stress is the Cch1/Mid1 channel. Taking into consideration that this response was largely absent in *mid1Δ* (Fig. 3B) it follows that Mid1 is essential for the DMSO‐induced entry of Ca^2+^ into the cytosol. At the same time, the faint response of *cch1Δ*, which might reflect a residual Mid1 activity (Fig. 3A) clearly indicates that the optimal channeling achieved by Mid1 requires the presence of intact Cch1.

DMSO is one of the most commonly used solvents in drug discovery and drug screening tests. Although highly effective as a drug carrier, DMSO itself imposes numerous challenges upon cell, including oxidative stress [50, 51]. This raises the question whether the Ca^2+^ pulses generated in the yeast cells by DMSO can mediate the activation of the general environmental stress response (ESR) program or rather induce a more specific response. It is indeed that Ca^2+^ can signal the occurrence of a variety of external stresses [12, 13, 14, 15, 16, 17, 18, 19, 20], but the Ca^2+^ waves generated by these stressors follow a monophasic pattern, regardless of the intensity or duration of the pulse. There are few studies that also reported a biphasic pattern for the Ca^2+^ waves, for example, in response to eugenol [52, 53], a natural compound that shares one structural resemblance with DMSO: amphiphilicity. Due to its amphiphilic traits, DMSO primarily interacts with the lipid bilayer; therefore, it is highly probable that before eliciting any oxidative stress, it imposes a mechanical stress on the cell membrane that can be signaled by the secondary messenger Ca^2+^ via the stretch‐activated Ca^2+^ channels. Mid1 is the only stretch‐activated channel described in yeast, and the lack of a DMSO‐induced [Ca^2+^]_cyt_ in the *mid1Δ* knockout cells (Fig. 3B) is a strong indication of Mid1's implication in the primary response to DMSO exposure. Considering the pattern of the [Ca^2+^]_cyt_ wave triggered by the DMSO exposure, the first phase likely represents the rapid Ca^2+^ entry through Mid1/Cch1 (caused also by any external stimulus), while the second phase may involve sustained Ca^2+^ influx facilitated by the mechanical (stretch) activation of Mid1. The biphasic response observed in *trpy1Δ*, although weaker, indicates that Trpy1 is not responsible for the second phase. Instead, its absence alters the overall dynamics, possibly due to changes in how the cell compensates for Ca^2+^ handling during stress episodes.

The yeast phosphoinositol pyrophosphate synthases Kcs1 and Vip1 were also shown to be involved in Ca^2+^‐related responses to DMSO exposure, since the knockout mutants *kcs1Δ* and *vip1Δ* were less tolerant to DMSO compared to the wild‐type (Fig. 1B). This was not surprising, considering the role of PP‐IPs in the response to various stressors [38, 39]. Interestingly though, *kcs1Δ* and *vip1Δ* display similarly deficient responses to DMSO regarding Ca^2+^ influx as *mid1Δ* and *cch1Δ*, respectively (Fig. 3A,B). While in *kcs1Δ* cells the response was totally absent (Fig. 4A), the *vip1Δ* displayed a very weak monophasic response to DMSO exposure (Fig. 4B).

## Conclusions

Collectively, our results suggest that the PP‐IPs whose synthesis is mediated by Kcs1 or Vip1 may have a role in activating Mid1/Cch1 (directly or indirectly), acting upstream to regulate the activity of Ca^2+^ channels. Notably, the lack of Ca^2+^ entry into the cytosol of *kcs1Δ* and *vip1Δ* cells could be seen only in the case of DMSO exposure, while [Ca^2+^]_cyt_ waves could still be recorded in response to other external stresses (e.g., alkaline, oxidative, Fig. 4C,D), indicating that Kcs1 and Vip1 may rather be involved in regulating cell response to mechanical stress. Such hypotheses are still difficult to defend, as long as there are not yet any reliable analytical means for the real‐time detection of PP‐IPs synthesis within live cells. The differential effects of *KCS1* and *VIP1* overexpression in cells exposed to DMSO, particularly in the *mid1Δ*, can be explained by their distinct roles in PP‐IP metabolism and their downstream impacts on cellular processes like Ca^2+^ homeostasis and stress responses. The overexpression of *KCS1* might disregard cellular processes by overproduction of 5PP‐IP5/5PP2‐IP4, increasing the sensibility to stressors, whereas the bifunctional role of *VIP1* (kinase and phosphatase [43]) could provide a protective role due to a finer tuning of PP‐IP levels under certain environmental conditions.

## Conflict of interest

The authors declare no conflict of interest.

## Author contributions

ICF conceived and designed the project. LIG, LLR, CVP, and SG acquired the data. LIG, LLR, CVP, and ICF analyzed and interpreted the data, and LIG and ICF wrote the paper.

## Data Availability

The data that support the findings of this study are available from the corresponding author (ileana.farcasanu@chimie.unibuc.ro) upon reasonable request.
